# Examining the Double-Edged Sword Effect of AI Usage on Work Engagement: The Moderating Role of Core Task Characteristics Substitution

**DOI:** 10.3390/bs15020206

**Published:** 2025-02-13

**Authors:** Xuan Liu, Yuxuan Li

**Affiliations:** School of Economics and Management, Nanjing Tech University, Nanjing 211816, China; xuanliu@njtech.edu.cn

**Keywords:** artificial intelligence usage, work engagement, core task characteristics substitution, double-edged sword effect test

## Abstract

As the application of artificial intelligence (AI) in the workplace increases, investigating its impact on work engagement is crucial for optimizing human resource management and enhancing organizational productivity and competitiveness. Based on the Conservation of Resources theory, this study investigated whether AI usage exhibits a double-edged sword effect on work engagement and examined the moderating role of core task characteristics substitution in this relationship. A two-wave study was conducted among 279 employees from China, and Hayes’s PROCESS macro was used to test the moderated mediation model. The findings indicated that (1) AI usage enhances work engagement by increasing psychological availability and indirectly increases work engagement by suppressing work alienation; (2) core task characteristics substitution diminishes the enhancing effect of AI usage on psychological availability and the inhibiting effect of AI usage on work alienation; and (3) overall, AI usage tends to suppress work alienation, demonstrating an empowering effect. However, under conditions of high core task characteristics substitution, AI usage can increase work alienation, revealing potential negative effects. The findings enrich our understanding of the complex impact of AI usage on work engagement and offer valuable insights for managers to improve employee experiences in the AI era.

## 1. Introduction

Artificial intelligence (AI) is considered one of the most revolutionary technologies (Brynjolfsson & McAfee, 2014; Charlwood & Guenole, 2022). According to a report by the Stanford Human-Centered Artificial Intelligence (Stanford HAI), the adoption rate of AI in businesses continues to grow: in 2023, 55% of companies started using AI tools, compared to 50% in 2022 and only 20% in 2017 (Maslej et al., 2024). In just six years, AI adoption has increased by 35 percentage points, reflecting the growing maturity of AI technology and companies’ recognition of its value. As noted by Von Krogh (2018), more and more organizations are introducing AI systems. This cutting-edge technology is gradually permeating many industries, including energy, finance, and healthcare, with organizations in these fields striving to explore how to effectively leverage AI to provide better services (L. Cao, 2022; Jiang et al., 2017; Lyu & Liu, 2021).

The positive effects of AI on business operations and production are becoming increasingly significant. AI can handle massive amounts of data, improve data accuracy, accelerate information transfer, increase information transparency, and help companies make more accurate decisions in a timely manner (Shang et al., 2024). Its capabilities in data analysis and pattern recognition drive the development of new products and innovation in services (Makridakis, 2017; S. Wang et al., 2024). In addition, AI can effectively release human resources, increase labor productivity, and save production costs by automating tasks and optimizing and adjusting workflows (Damioli et al., 2021; Graetz & Michaels, 2018; C.-H. Yang, 2022). However, widely using AI also introduces a series of potential negative impacts. When a large number of repetitive tasks are replaced by machines, a subsequent decline in employee wage levels and even job losses follow (Acemoglu & Restrepo, 2020; Arntz et al., 2016). AI’s automation capabilities will increase the demand for skilled workers with technical expertise in firms while limiting employment opportunities and income levels for unskilled workers, potentially exacerbating income inequality (Autor et al., 2003). At the same time, because AI requires large amounts of data for training and learning, employees as well as businesses are at an increased risk of information leakage and security vulnerability, which poses a threat to the management of the business (Vrontis et al., 2022). Bias and misinformation-based challenges in AI systems can also exacerbate certain dysfunctions in organizations (Malik et al., 2021). These adverse effects resulting from the application of AI technology undoubtedly present challenges for both businesses and society.

In the field of organizational behavior, to determine the positive or negative impacts of AI on employees, researchers are extensively investigating how AI usage in the workplace affects employees’ psychological states and work attitudes. On the one hand, the application of AI facilitates teleworking and flexible working, helping employees achieve a better work–life balance and enhancing job satisfaction and loyalty (Malik et al., 2021). The distinct form of agentic rationality enables AI to achieve levels comparable to or even surpassing humans in performing cognitive tasks (Murray et al., 2021). For instance, because AI-driven decisions focus solely on the facts of an event without emotional bias, they help employees make more informed choices (Khanzode & Sarode, 2020). On the other hand, the drastic changes brought about by digital transformation trigger psychological unease among employees (Malik et al., 2021). AI makes employees’ work overload as well as facing the need to update their skills, both of which contribute to potential sources of job stress (Paluch et al., 2022). This stress contributes to various negative outcomes, such as increased turnover intention, reduced job satisfaction, and heightened job insecurity among employees (Bhargava et al., 2021; He et al., 2023; J. Li et al., 2019). Therefore, there are two-sided effects of AI on employees’ work psychology and behavior.

Work engagement, as a work attitude, is defined as the degree to which organizational members are integrated with their work roles, and the physical, emotional, and psychological resources possessed by the individual are necessary prerequisites for work engagement (Kahn, 1990). Employee work engagement is related to their attitudes, intentions, and behaviors (Saks, 2006). Higher work engagement helps employees to maintain good mental health (Tisu et al., 2020), stimulates greater enthusiasm in their work, thereby enhancing job performance (Tisu et al., 2020), increasing organization commitment (Hu & Schaufeli, 2011), and reducing turnover intention (Schaufeli & Bakker, 2004). At the same time, work engagement enhances customer loyalty by shaping a positive service climate (Salanova et al., 2005), which positively affects organizational productivity, Innovativeness and competitiveness. Therefore, work engagement is not only about the psychological state of individual employees but also a key factor in the success of the team and the entire organization. Work engagement has been a focal issue in academia. Existing studies have mainly explored antecedent variables that influence work engagement, including organization support, leadership styles, work environment, and interpersonal relationships (Jung et al., 2021; Lee et al., 2024; Ly, 2024; Oh et al., 2018). However, fewer studies have examined how AI usage impacts work engagement.

According to Conservation of Resources (COR) theory, anything that holds value to someone can be considered a resource. Individuals always try to acquire and maintain the resources they consider valuable, including material resources, energy resources, conditional resources, and personal resources (Hobfoll, 1989). Employees will have a positive psychological experience when they acquire resources, while employees will have a negative psychological experience when they feel resource depletion (Hobfoll, 1989). On the one hand, AI aids employees in reducing resource depletion by simplifying repetitive tasks and offering personalized assistance. At the same time, AI is also effective in enhancing individuals’ sense of resource acquisition by helping them tackle complex problems. This sense of resource abundance translates into psychological confidence and readiness, known as psychological availability. The core perspective of COR theory is that when individuals have sufficient resources, they are able to effectively reduce the consumption of resources at work and are more able to acquire resources. Individuals also tend to actively engage in role tasks when they feel resource-rich (Hobfoll et al., 2018), stimulating their work engagement. On the other hand, employees may perceive AI as a threat to resources due to AI substitution pressure, gradually alienated interpersonal relationships, and ambiguous professional roles (Benbya et al., 2020; Goto, 2021; Tang et al., 2023), which may lead to severe resource depletion. At this point, employees may develop a sense of work alienation due to resource loss. In order to protect their remaining resources, they will reduce their interactions with the organization and lower their work engagement (Hirschfeld et al., 2000). Thus, the usage of AI may have a double-edged sword effect on work engagement, with work alienation and psychological availability potentially playing mediating roles in the relationship between AI usage and work engagement.

Currently, extensive research on human–computer collaboration is being conducted in academia. Established studies have mainly explored the relationship between humans and robots from the perspectives of task allocation (Müller et al., 2017; L. Wang et al., 2020), proximity (Sowa et al., 2021), and operational safety (Matheson et al., 2019). Recent research in the field of organizational behavior has further explored various factors influencing employees’ psychological and behavioral responses in human–AI collaboration. For instance, personality traits (Wu et al., 2024), technological design features (Peng et al., 2024), and leadership styles (Yin et al., 2024) all play significant roles in shaping this process. As employees are experiencing the substitution of traditional job tasks and the reconfiguration of job characteristics in the context of the widespread use of AI technologies (Verma & Singh, 2022). Consequently, some scholars have begun to investigate the role of job characteristics in human–AI collaboration (Berretta et al., 2023; Coupé, 2019), but no research has yet focused on the impact of this change on workplace employees. Based on this, this study proposes a novel variable according to the job characteristics model (JCM), core task characteristics substitution (CTCS). We define core task characteristics substitution as the extent to which core task characteristics, such as autonomy, task identity, and significance, in individual work tasks are replaced by AI. When employees use AI, and their core task characteristics are highly substituted by AI, it will diminish the meaning and significance of their work, thereby increasing work alienation and reducing work engagement. Conversely, when the substitution of core task characteristics by AI is minimal, AI can serve as a supportive tool, helping employees handle mundane and repetitive tasks more efficiently. This conserves employees’ psychological resources, enhances psychological availability, and ultimately increases their work engagement. Thus, core task characteristics substitution may moderate the mediating roles of work alienation and psychological availability.

In summary, this study introduces work alienation and psychological availability as mediating variables to examine whether AI usage has a double-edged sword effect on work engagement. Moreover, it incorporates core task characteristics substitution as a moderating variable to systematically understand the underlying mechanisms and boundary conditions of AI’s impact on work engagement. This research makes four main contributions to the literature. First, it helps to identify the factors influencing work engagement in the context of AI usage, thereby enriching the antecedent variables of work engagement. Second, this study aids in clarifying the positive and negative mechanisms of AI usage, deepening the understanding of AI-related variables, and thus enriching COR theory. Third, this study contributes to understanding the boundaries within which the positive and negative effects of AI usage operate, clarifying the task substitution attributes of AI and their mechanisms, thereby improving the JCM in the context of AI. Fourth, this study also provides theoretical and practical guidance for managers to develop effective strategies to maximize work engagement and mitigate negative effects.

## 2. Theoretical Background and Research Hypothesis

### 2.1. The Mediating Role of Psychological Availability

An individual’s psychological availability, as a psychological condition, is defined as the perceived availability of physical, emotional, or cognitive resources at a specific moment (Kahn, 1990). Psychological availability reflects the level and state of confidence held by an individual in meeting the demands involved in the job (Vinarski-Peretz & Carmeli, 2011), i.e., an individual’s belief that he or she possesses the confidence to fully mobilize and effectively utilize these resources in order to meet the challenges of the job. The fundamental assumption of COR theory is that people are always actively striving to conserve, protect, and acquire resources they deem valuable. The number of resources an individual possesses and their perception of resource availability influence their level of psychological availability (Chikoko et al., 2014).

This study argues that AI usage enhances employees’ psychological availability. First of all, the use of AI technology helps to reduce the burden on employees in performing mechanical and repetitive tasks, freeing them from simple and repetitive labor (Marinova et al., 2016). This liberation of psychological resources allows employees to have more autonomy and creativity in their work patterns and content (Makridakis, 2017; Malik et al., 2021). Under these conditions, AI reduces work fatigue and boosts job satisfaction and motivation, thereby enhancing psychological availability.

Second, AI systems with advanced algorithms and robust data processing capabilities (Shang et al., 2024) enable the rapid extraction of key information, providing employees with timely and useful data to make more accurate and efficient decisions. For example, Theis et al. (2014) pointed out that the application of AI in Augmented Reality (AR) technology can effectively filter out unnecessary information, providing only the necessary and precise information. This reduces cognitive load during complex and high-intensity tasks. Cognitive stress, as a hindrance stressor, consumes an individual’s limited physiological and psychological resources (Lepine et al., 2005). Therefore, when stress and anxiety are reduced, employees do not need to expend excessive psychological energy to cope with stressors, thereby conserving their psychological resources and increasing their psychological availability (Binyamin & Carmeli, 2010), enabling them to remain productive and focused in the face of difficult problems.

Furthermore, given that AI technology continuously creates new work models, employees need to “reskill” to adapt to the new work content when collaborating with AI (Zirar et al., 2023). This means that employees must learn new skills, upgrade their existing skills, or transition to entirely new skill areas to boost their employability and occupational competitiveness. Proper use and integration of AI in the workplace not only help employees overcome technological challenges but also enhance their capabilities and skills. For example, Shanmugalingam et al. (2020) argue that gamification mechanisms in AI tools enhance employees’ intrinsic and extrinsic motivation, while predictive analytics offer personalized feedback and rewards, promoting learning and development. Brynjolfsson et al. (2023) also found that adoption of suggestions provided by generative AI significantly contributes to continuous skill enhancement among workers. This “reskilling” helps employees to adapt to the new environment of the AI era in a timely manner, which improves their career adaptability and increases their confidence in their future career development (Kong et al., 2021). Psychological availability is precisely the reflection of an individual’s confidence and state of readiness in meeting the demands involved in their work (Vinarski-Peretz & Carmeli, 2011). Therefore, by enhancing employees’ skills, AI helps them participate in work more confidently, making them more assured of their abilities and status, and thus increasing their psychological availability.

The COR theory holds that when individuals possess sufficient resources, they are able to effectively reduce the consumption of resources by their work and are better equipped to acquire additional resources. Individuals are more likely to engage positively in their role tasks when they feel resource-rich (Hobfoll et al., 2018), thereby stimulating their work engagement. Since work engagement measures employees’ enthusiasm and concentration in their work, it represents their positive emotional and cognitive states (Schaufeli et al., 2006). Whereas higher psychological availability means that employees perceive a greater sense of psychological readiness and are willing to actively and proactively engage in creative activities, thus increasing their work engagement (Binyamin & Carmeli, 2010; Russo et al., 2016). Research by May et al. (2004) has confirmed that psychological availability is one of the three psychological conditions that influence whether and how individuals engage in their roles. Schaufeli and Salanova (2007) also pointed out that work engagement is a reflection of psychological availability. Therefore, this study argues that when employees use AI in their work, AI empowers them, saves and supplements the individual’s psychological resources, thereby enhancing their psychological availability and, in turn, further increasing their work engagement. In summary, this study proposes the following research hypotheses:

**H1.**  *AI usage positively influences psychological availability.*

**H2.**  *Psychological availability positively influences work engagement.*

**H3.**  *Psychological availability mediates the relationship between AI usage and work engagement.*

### 2.2. The Mediating Role of Work Alienation

In previous studies on alienation, Seeman (1959) first proposed that there are five elements that can lead to alienation, which are powerlessness, meaninglessness, normlessness, isolation, and self-estrangement. As social and organizational psychology have evolved, scholars have applied the traditional concept of alienation to the workplace context, defining work alienation as a psychological state in which employees feel separated from their work due to unmet needs or discrepancies between their expectations and the actual work environment (Banai et al., 2004). The COR theory posits that individuals always strive to acquire and maintain the resources they consider valuable, including material, energetic, conditioned, and individual resources. Additionally, the potential or actual loss of these resources constitutes a threat to them. Social relationships, the degree of involvement in decision-making, autonomy, opportunities for career development, social support, an optimistic personality, and rewards can all be considered valuable resources by individuals (X. Cao & Qu, 2014). When individuals perceive that resources are expected or have failed to be rewarded, they will feel stress and produce a series of negative psychological and physiological responses. Work alienation precisely reflects the negative experiences of employees feeling psychologically detached and behaviorally distant from their work and the workplace (Zhou & Long, 2011).

This study suggests that AI usage may contribute to work alienation among employees. First, widespread technology use can negatively affect human face-to-face communication, leading to distraction in socializing and reducing the likelihood of deeper communication (Drago, 2015). Communication, as a “help” (Itoh, 1991) or “information subsidy” (Hall & Deardorff, 2006) activity, provides necessary information related to decision-making and helps the recipient complete tasks more efficiently. In organizations, face-to-face communication is essential to enhance the effectiveness of mutual support among colleagues (Battiston et al., 2020). However, with the deeper use of AI systems, employees are increasingly relying on machines rather than human coworkers for collaboration and feedback, weakening the individual’s ability to interact interpersonally and emotionally (Tang et al., 2023). And negative interpersonal relationships often lead to high levels of work alienation (Nair & Vohra, 2010), an important cause of alienation formation.

Second, compared to traditional AI that follows human-preset rules, AI systems that generate rules based on big data analysis are often difficult for humans to understand (Liu, 2021). Employee concerns about AI accuracy (Jain et al., 2018), data privacy and security (Przegalinska et al., 2019), and transparency of algorithmic systems (Laumer et al., 2019) hinder full trust in AI. Especially with the emergence of AI capable of making decisions independently, AI systems have been able to perform tasks and workflows without any human involvement (Benbya et al., 2020). Human creativity is now being taken over by AI, and employees are no longer directly involved in the decision-making process, which diminishes the decision-making power and autonomy of humans in organizations (Holford, 2019). Work alienation occurs when employees feel that their sense of control over their work has been taken away from them and that work has become a mechanical activity carried out solely to address external needs (e.g., money, security, etc.) that do not express their unique abilities and potential (Kanungo, 1983).

Finally, AI systems can quickly and efficiently perform many tasks that would otherwise be the responsibility of humans, and employees may find their roles in the workplace being simplified or even replaced, posing unprecedented challenges to their professional role identity (Strich et al., 2021). Professional role identity is an individual’s self-definition as a member of a particular profession (Chreim et al., 2007), involving how they understand their role, responsibilities, and status within the professional environment, and the alignment of this role with their personal values, goals, and beliefs (Ashforth, 2001). In the AI era, employees need to redefine their roles in the workplace, as traditional roles may no longer be applicable. The lack of real-world AI usage examples can hinder the formation of new role identities, leading to a sense of ambiguity in their professional roles (Goto, 2021). The uncertainty created by role ambiguity not only diminishes an individual’s expectations of performance achieved through effort and the relationship between that performance and the rewards received (Jackson & Schuler, 1985), but also diminishes the employee’s sense of control over the outcome, which in turn induces feelings of helplessness (Tayfur, 2012). Employees then feel powerless and lose meaning in their work, which breeds feelings of alienation at work (Kahn, 1990; Seeman, 1967).

Therefore, AI usage will increase employees’ sense of work alienation, which in turn will reduce their work engagement. In COR theory, when facing resource loss, individuals tend to take immediate action to prevent further loss (Cheek & Buss, 1981), minimizing damage. That is, when employees perceive that AI has led to a loss of individual resources, they may reduce their work engagement to prevent further resource depletion (Hobfoll, 2002). Existing studies generally support the idea that work alienation significantly reduces work engagement. For example, Hirschfeld et al. (2000) argue that when employees experience work alienation, they tend to separate their personal identity from their work activities, gradually losing enthusiasm and focus for their work, making it difficult to stimulate intrinsic motivation and initiative, thus reducing their interaction with the organization and decreasing work engagement. Additionally, employees with a sense of work alienation often have lower identification with the organization’s goals and values, are less willing to put in extra effort to achieve organizational objectives, and this exacerbates the decline in their organizational commitment, further reducing their work engagement (Becker, 1960). Therefore, this study posits that while the use of AI in the workplace may simplify tasks, it can also lead to the loss of important resources for individuals, thereby increasing their sense of work alienation. After experiencing work alienation, individuals may reduce their work engagement to avoid further resource depletion. In summary, this study proposes the following research hypotheses:

**H4.**  *AI usage positively influences work alienation.*

**H5.**  *Work alienation negatively influences work engagement.*

**H6.**  *Work alienation mediates the relationship between AI usage and work engagement.*

### 2.3. Moderating Effect of Core Task Characteristics Substitution

In the face of the substitution crisis brought about by the increasing maturity of AI technology, academia is actively exploring how to optimize and promote collaboration between humans and machines.

In studies on human–machine collaboration, the relationship between humans and robots can be categorized into human–robot coexistence, human–robot interaction (HRI), human–robot cooperation, and human–robot collaboration from the perspective of task allocation (Müller et al., 2017; L. Wang et al., 2020). From the perspective of proximity, these relationships can be classified as competing or working separately, supplementing each other, interdependent on each other, and hybrid of the two (Sowa et al., 2021). In terms of operational safety, human–robot collaboration models can be divided into safety-rated monitored stop (SMS), hand guiding (HG), speed and separation monitoring (SSM), and power and force limiting (PFL) (Matheson et al., 2019). In the field of organizational behavior, recent studies have extensively investigated various factors influencing employees’ psychological responses and behaviors in human–robot collaboration scenarios. Research indicates that key elements such as personality traits (Wu et al., 2024), technological design features (Peng et al., 2024), and leadership styles (Yin et al., 2024) are increasingly recognized for their significant roles in this process. As AI programs continue to develop and upgrade, AI systems are gradually becoming capable of autonomously taking over entire workflows rather than just specific tasks (Bailey et al., 2019). Employees are experiencing the substitution of traditional job tasks and the redefinition of job characteristics (Verma & Singh, 2022), and these systems can even replace the core workflows of an entire occupation.

According to the job characteristics model, the job itself is a way to motivate employees, and this motivation depends on the characteristics of the work itself and the employee’s subjective perception of it. The five core dimensions of this model—skill variety, task identity, task significance, autonomy, and feedback (Hackman & Oldham, 1976)—can, when optimized and combined, prompt employees to undergo a psychological transformation process, leading to what are known as “critical psychological states”: experienced meaningfulness of the work, responsibility for outcomes, and knowledge of the results. These critical psychological states ultimately influence an individual’s job satisfaction, motivation levels, and, in turn, their job performance. In the era of artificial intelligence, these five core dimensions will undergo changes:

Skill variety. The widespread adoption of AI is likely to exacerbate skill polarization in the labor market, enhancing skill variety in high-skilled occupations while having adverse effects on other types of jobs (Holm & Lorenz, 2022). The division of labor becomes more refined due to AI (Lobova et al., 2020), and as Adam Smith noted in “The Wealth of Nations”, the division of labor allows employees to focus on a specific production stage or task, promoting skill specialization. This also means that employees are concentrated on a narrower range of work, leading to a reduction in skill variety.

Task identity. As AI segments workflows and automates multiple steps, employees responsible for only part of the process may find it difficult to see the full picture of their work and its ultimate impact, thereby reducing the perceived integrity of the task.

Task significance. If important tasks are performed by the AI, employees may not perceive the significance of their contribution.

Autonomy. During times of technological transition, workers are often perceived to lack autonomy or a sense of control (Nazareno & Schiff, 2021). When AI systems directly issue operational instructions to employees or control the workflow, AI takes on a direct controlling role, reducing employees’ control over their tasks and decision-making space, thereby diminishing their autonomy at work (Holm & Lorenz, 2022).

Feedback. Although, in theory, artificial intelligence can provide more immediate feedback compared to human managers (Porter & Grippa, 2020), this feedback is often based on predefined standards and algorithmic logic, lacking humanistic considerations.

Based on this, we consider human–machine collaboration from the perspective of job characteristics and propose a new variable: core task characteristics substitution. Core task characteristics substitution refers to the extent to which core task characteristics, such as autonomy, task identity, and significance, in an individual’s work tasks are replaced by AI. It can moderate the relationship between AI usage and work alienation, as well as the relationship between AI usage and psychological availability. If AI replaces unimportant job characteristics and takes on simple, repetitive, tedious, and boring tasks, it can reduce employees’ physical and mental fatigue (Qiu et al., 2022), decrease the depletion of their physical and psychological resources, and thus increase psychological availability. At the same time, employees can focus on work that requires more creativity and strategic planning (Makridakis, 2017; Malik et al., 2021), making their work meaningful (Bankins & Formosa, 2023), increasing psychological resources, and thus enhancing psychological availability.

On the contrary, when AI is increasingly applied in the workplace and replaces core task characteristics such as meaningfulness and task integrity, it can lead to employees experiencing work alienation. The acquisition of a sense of meaning is very important to individuals (Kotter-Grühn et al., 2009), and it not only relates to personal well-being and job satisfaction but also enhances employees’ creativity and loyalty to the organization (Sadeghian & Hassenzahl, 2022). Therefore, employees should be more involved in work they find meaningful (Fairlie, 2011). However, if employees lose their creativity in AI-collaborative work, they will be unable to engage in activities that were originally rich in meaning (Parmer, 2023). When meaningful work is replaced by AI and new mundane tasks are created, along with restrictions on worker autonomy and unfair distribution of benefits, employees’ sense of meaning at work can be diminished (Bankins & Formosa, 2023). The resulting feelings of powerlessness and meaninglessness are sources of work alienation (Seeman, 1959). Based on this, the following research hypotheses are proposed in this study:

**H7.**  *Core task characteristics substitution plays a moderating role between AI usage and psychological availability. The positive impact of AI usage on psychological availability will weaken when more core task characteristics are substituted.*

**H8.**  *Core task characteristics substitution plays a moderating role between AI usage and work alienation. The positive impact of AI usage on work alienation will be enhanced when more core task characteristics are substituted.*

According to the COR theory, the same situation can be perceived either as a depletion of existing resources or as a unique opportunity to acquire new resources, depending on the perspective (Halbesleben et al., 2014; Hobfoll et al., 2018). As mentioned earlier, in the context of widespread AI technology adoption, employees’ different perceptions of resource depletion and acquisition may trigger a sense of work alienation or enhance psychological availability. Whether AI usage has a positive or negative impact depends crucially on the degree of core task characteristics substitution, which in turn further affects their work engagement. Therefore, for employees with lower core tasks being substituted, AI primarily plays a supportive role, helping to address simple and repetitive tasks. This allows employees to save time and energy for more meaningful work, enhancing their psychological availability and making them more willing to actively participate in work activities, thus increasing their work engagement. This phenomenon exemplifies the gain spiral within COR theory: when individuals possess sufficient valuable resources, they are not only more capable of acquiring additional resources, but also these newly acquired resources further facilitate even more resource growth. This creates a virtuous cycle. Conversely, compared to employees with less core task characteristics substitution, those with more core tasks being substituted are more likely to lose a sense of meaning and enjoyment in their work, experiencing severe resource depletion, which leads to greater work alienation. To avoid further loss of resources, these employees may reduce their work engagement. This situation aligns with the loss spiral in COR theory: once individuals begin to experience resource loss, they tend to take measures to prevent further loss. However, these defensive behaviors can paradoxically lead to even more resource depletion, creating a vicious cycle. Based on this, this study proposes the following research hypotheses:

**H9.**  *Core task characteristics substitution moderates the mediating role of psychological availability in the relationship between AI usage and work engagement.*

**H10.**  *Core task characteristics substitution moderates the mediating role of work alienation in the relationship between AI usage and work engagement.*

**Figure 1:**
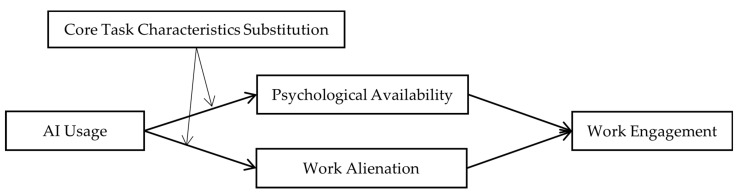
Theoretical model.

In summary, the theoretical model of this study is shown in Figure 1.

## 3. Method

### 3.1. Sample and Procedure

The Chinese online platform Credamo, similar to MTurk, was utilized to collect data in this study. When distributing the questionnaire, we set specific target sample criteria to ensure that only eligible participants received the survey. Given our focus on high-level AI usage areas, we primarily targeted knowledge workers in industries including information technology services and the intelligent manufacturing industry. Compared to other data collection methods, online platforms offer a more diverse sample pool and flexible questionnaire design features, facilitating the acquisition of high-quality data. Prior to the formal survey, a pilot study was conducted, which included two screening questions: (1) a choice question asking, “Do you use AI in your work?”; (2) an open-ended question requesting, “Please describe how you use AI in your work”. Responses indicating “No” to the choice question automatically ended the survey, while the open-ended responses were reviewed by researchers before participants could proceed to the formal survey. In order to reduce the effect of common method bias on the relationship between variables, the research data were collected at two distinct time points (with a half-month interval). At Time 1, employees reported AI usage, core task characteristics substitution, and demographic variables (gender, age, educational level, and work experience). Out of 339 distributed questionnaires, 306 valid responses were collected. At Time 2, the same employees reported work alienation, psychological availability, and work engagement. A total of 306 questionnaires were distributed; after excluding responses with clear patterns or mismatched answers, 279 valid questionnaires were obtained, resulting in an effective response rate of 82.3%.

The sample features are as follows: regarding gender, there are 153 males (54.8%) and 126 females (45.2%); the average age of the sample is 31.54 years (SD = 8.30); for education level, 46 people (16.5%) are college graduates or below, 160 people (57.3%) are undergraduates, and 73 people (26.2%) are masters and above; in terms of the number of years of working experience in the organization, 41 people (14.7%) have worked in the organization for less than one year, 100 people (35.8%) have worked there for 1–5 years, and 64 people (22.9%) have worked there for 6–10 years, and 41 people (14.7%) have worked there for 11–15 years, and 33 people (11.8%) have worked more than 15 years.

### 3.2. Measure

The measurement items for the main variables involved in this study were based on well-established scales that had been widely used and validated by scholars. Since the subjects of this study were employees of Chinese companies, a strict translation-back translation procedure was followed to ensure the questionnaire’s accuracy. All items used a five-point Likert-type scale ranging from 1 = ‘Strongly disagree’ to 5 = ‘Strongly agree’.

Artificial intelligence usage. AI usage was assessed by a three-item scale developed by Tang et al. (2022). An example item was: “I used artificial intelligence to carry out most of my job functions”. The Cronbach’s alpha for the scale was 0.882.

Core task characteristics substitution. In constructing the measurement tool for evaluating the substitution of core task characteristics, we drew upon Hackman and Oldham’s job characteristics model (JCM) from 1976, which categorizes job characteristics into five core dimensions. Based on the needs of AI usage scenarios, this study designed a five-item scale derived from these dimensions to assess the extent to which AI usage substitutes for core task characteristics. According to the JCM definitions, we provided detailed explanations of each job characteristic before posing the questions. For instance, regarding “job autonomy”, we defined it as the extent to which employees determine the pace, methods, and sequence while accomplishing tasks. An example item was: “To what extent has the artificial intelligence used in your work reduced your job autonomy?” Higher scores indicate a greater degree to which AI usage has substituted for individual core job characteristics. Cronbach’s alpha for the scale was 0.887.

Psychological availability. Psychological availability was evaluated by a five-item scale developed by May et al. (2004). An example item was: “l am confident in my ability to handle competing demands at work”. Cronbach’s alpha for the scale was 0.743.

Work alienation. Work alienation was evaluated by an eight-item scale developed by Nair and Vohra (2010). An example item was: “I often wish I were doing something else”. The Cronbach’s alpha for the scale was 0.918.

Work engagement. Work engagement was assessed by a nine-item scale developed by Schaufeli et al. (2006). An example item was: “I am enthusiastic about my job”. The Cronbach’s alpha for the scale was 0.857.

Control variables. Referring to related studies on work engagement by Abboushi (1990), Rabinowitz and Hall (1977), and Rabinowitz et al. (1977), this study sets gender, age, education level, and work experience as control variables.

### 3.3. Analytical Method

This study employed SPSS 21.0 and Mplus 8.3 for data analysis. First, Mplus 8.3 was used for confirmatory factor analysis and the assessment of common method bias. Next, SPSS 21.0 was utilized for Harman’s single-factor test, descriptive statistics, correlation analysis, and hierarchical regression analysis. Finally, the Process 3.2 macro was used to examine both mediation effects and moderated mediation effects. Data will be included if it meets our recruitment requirements. Exclusion criteria include cases with excessive missing values, identical responses to all questions, and inconsistent answers across related items.

## 4. Results

### 4.1. Confirmatory Factor Analysis

Before testing the hypotheses, we conducted a confirmatory factor analysis (CFA) to test the discriminant validity of five latent variables: AI usage, core task characteristics substitution, psychological availability, work alienation, and work engagement. As shown in the results of Table 1, the fit index of the five-factor model was significantly better than the other models with the best fit (*χ*^2^ = 866.736, *df* = 424, CFI = 0.900, TLI = 0.891, RMSEA = 0.061, SRMR = 0.063), which indicated that the five variables in this study had good discriminant validity.

### 4.2. Common Method Bias

In order to control common method bias, this study used the Harman single-factor test for validation. The results of the unrotated exploratory factor analysis showed that the first principal component explained 26.406% of the total variance, which was below the threshold of 40% (Podsakoff et al., 2003). Additionally, this study also employed the method of adding an unmeasured latent factor to test for common method bias. The results indicated that, compared to the five-factor model, the fit indices of the six-factor model did not show significant improvement (ΔCFI = 0.009, ΔTLI = 0.011, ΔRMSEA = 0.007, ΔSRMR = 0.008). Therefore, common method bias did not pose a threat to the findings of this study.

### 4.3. Descriptive Statistical Analysis

The means, standard deviations, correlation coefficients, and their significance levels for all variables are shown in Table 2. The usage of AI was positively related to psychological availability (r = 0.254, *p* < 0.01) and negatively related to work alienation (r = −0.188, *p* < 0.01), which was inconsistent with our hypothesis and would be further analyzed subsequently. Work engagement was positively related to psychological availability (r = 0.357, *p* < 0.01) and negatively related to work alienation (r = −0.387, *p* < 0.01), providing preliminary support for the research hypotheses.

### 4.4. Hypothesis Testing

#### 4.4.1. Mediating Effect Testing

As shown in Table 3, the usage of AI had a significant positive effect on psychological availability (*β* = 0.265, *p* < 0.001, Model 4), thus supporting H1. It also had a significant negative effect on work alienation (*β* = −0.182, *p* < 0.01, Model 1), which was inconsistent with the original hypothesis. Instead of increasing work alienation, the usage of AI suppressed it, so H4 was not supported. As shown in Model 9, when AI usage, work alienation, and psychological availability were included in the equation simultaneously, psychological availability positively influenced work engagement (*β* = 0.213, *p* < 0.001), and work alienation negatively influenced work engagement (*β* = −0.317, *p* < 0.001). These results indicated that AI usage positively impacts work engagement through two pathways: by enhancing psychological availability and by reducing work alienation. Psychological availability and work alienation mediated the relationship between AI usage and work engagement. The usage of AI demonstrated positive empowering effects through both pathways, and no negative effects of AI were found. Therefore, H2, H3, and H5 were supported, and H6 was partially supported.

To further verify the significance of the mediating effect, bootstrapping was conducted with 5000 replicates (Preacher & Hayes, 2008). The results of the indirect effect analysis were shown in Table 4. The indirect effect of AI usage on work engagement through psychological availability was 0.032, with a 95% confidence interval of [0.012, 0.059], which did not include 0, indicating that psychological availability played a mediating role, thus further supporting H3. The indirect effect of AI usage on work engagement through work alienation was also 0.033, with a 95% confidence interval of [0.011, 0.065], which also did not include 0, indicating that work alienation played a mediating role, thus further supporting H6.

#### 4.4.2. Moderating Effect Testing

This study used hierarchical regression analysis to examine the moderating effects, and the results were shown in Table 3. The results indicated that the interaction term between AI usage and core task characteristics substitution significantly predicted psychological availability (*β* = −0.157, *p* < 0.01) and work alienation (*β* = 0.300, *p* < 0.001). This suggested that the moderating effects indeed existed, thus supporting H7 and H8.

**Figure 2:**
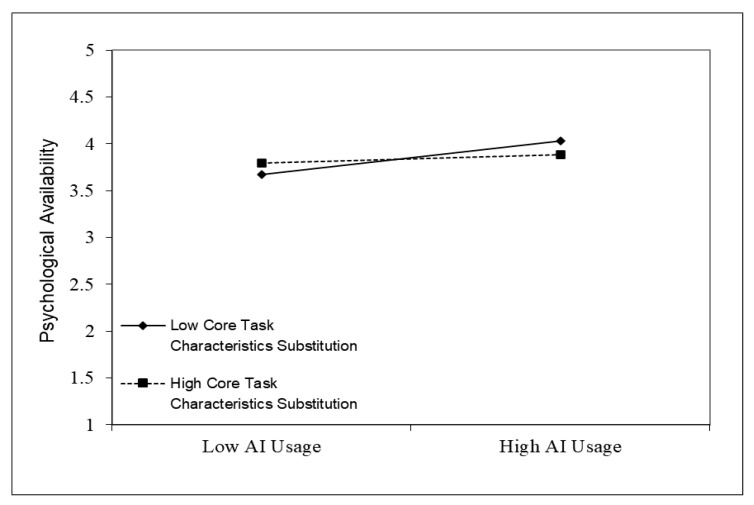
The moderating effect of core task characteristics substitution on AI usage and psychological availability.

To further illustrate the impact of different levels of core task characteristics substitution on these relationships, the simple slopes analysis was applied to provide graphs of the moderating impact (Aiken & West, 1991). As shown in Figure 2, when core task characteristics substitution was low (M − 1SD), the effect of AI usage on psychological availability was significant (simple slope = 0.181, t = 4.047, *p* < 0.001). However, when core task characteristics substitution was high (M + 1SD), the effect of AI usage on psychological availability was not significant (simple slope = 0.043, t = 0.962, *p* > 0.05). The analysis results indicated that AI usage can significantly enhance employees’ psychological availability only when core task characteristics substitution was low, thus partially supporting H7.

**Figure 3:**
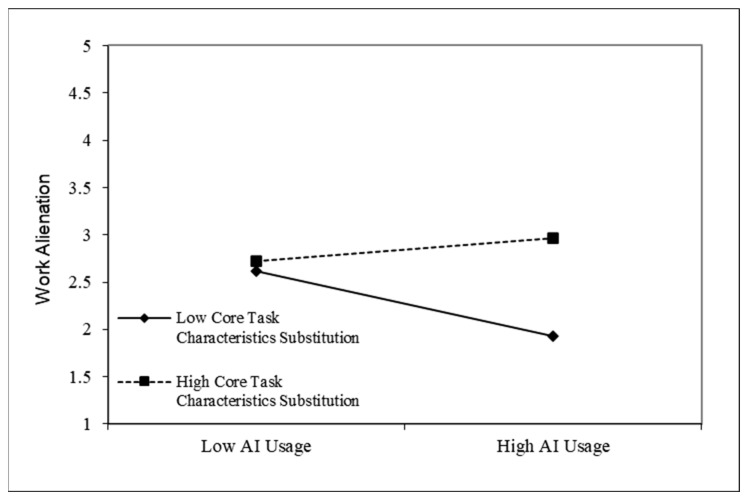
The moderating effect of core task characteristics substitution on AI usage and work alienation.

As shown in Figure 3, when core task characteristics substitution was low (M − 1SD), the regression slope of AI usage on work alienation was negative and significant (simple slope = −0.400, t = −5.657, *p* < 0.001). This meant that under conditions of low core task characteristics substitution, AI usage had a significant negative predictive effect on work alienation. When core task characteristics substitution was high (M + 1SD), the regression slope of AI usage on work alienation was positive and significant (simple slope = 0.144, t = 2.036, *p* < 0.05). This indicated that under conditions of high core task characteristics substitution, AI usage had a significant positive predictive effect on work alienation. This partially confirmed the correctness of the original hypothesis. Although overall, AI usage tends to inhibit and reduce work alienation, demonstrating its empowering role, in situations where core task characteristics were substituted, AI usage can increase employees’ work alienation.

#### 4.4.3. Moderated Mediation Effects Testing

As shown in Table 5, in the path mediated by psychological availability, when core task characteristics substitution was low (M − 1SD), the indirect effect of AI usage on work engagement through psychological availability was 0.047, with a 95% CI [0.018, 0.079], which did not include 0; whereas when core task characteristics substitution was high (M + 1SD), the indirect effect of AI usage on work engagement through psychological availability was 0.012, with a 95% CI [−0.006, 0.043], which includes 0. The difference in indirect effects between the two groups was −0.035, with a 95% CI [−0.065, −0.004], which did not include 0, indicating a significant difference. This suggested that core task characteristics substitution had moderated the indirect path from AI usage to work engagement via psychological availability. Therefore, H9 was supported.

In the path mediated by work alienation, when core task characteristics substitution was low (M − 1SD), the indirect effect of AI usage on work engagement through work alienation was 0.078, with a 95% CI [0.040, 0.124], which did not include 0. When core task characteristics substitution was high (M + 1SD), the indirect effect of AI usage on work engagement through work alienation was −0.022, with a 95% CI [−0.054, 0.013], which includes 0 and was not significant. The difference in indirect effects between the two groups was −0.101, with a 95% CI [−0.157, −0.048], which did not include 0, indicating a significant difference. This suggested that core task characteristics substitution had moderated the indirect path from AI usage to work engagement via work alienation. Thus, H10 was supported.

## 5. Discussion

According to the COR theory, this study examined the impact of AI usage on work engagement. Through theoretical research and empirical analysis, the results indicate that:

First, psychological availability plays a significant mediating role between AI usage and work engagement. AI usage can enhance individuals’ psychological availability, which in turn increases work engagement.

Second, work alienation also plays a significant mediating role between AI usage and work engagement. AI usage reduces individuals’ work alienation, thereby weakening the negative impact of work alienation on work engagement and indirectly enhancing work engagement. Contrary to our initial hypothesis that AI usage would increase work alienation, statistical results show that AI usage increases work alienation only under conditions of high core task characteristics substitution. However, overall, AI usage tends to inhibit work alienation. Possible reasons for this are as follows: Currently, AI has been widely applied in many fields in China, including management practices, but most domestic enterprises’ human–AI collaboration is still at an early stage, primarily in a supportive role. AI was initially designed to assist humans in completing tasks rather than completely replacing them (Zirar et al., 2023). AI is more often used to help employees with simple and repetitive tasks, playing a supportive role without significantly substituting core tasks. Most domestic studies also indicate that the empowering effects of current AI usage far outweigh their negative impacts, enabling psychological empowerment and enhancing career development capabilities (Huang et al., 2024; Z. Li et al., 2023). Additionally, existing research has found that AI usage does not necessarily weaken employees’ interpersonal collaboration and communication skills. Porter and Grippa (2020) point out that AI, by providing immediate and personalized feedback, promotes collaboration and communication among team members, thus becoming a key factor in overcoming the challenges of remote work. This enhanced social interaction helps build stronger team cohesion and a sense of belonging, thereby reducing work alienation.

Third, core task characteristics substitution has a significant moderating effect on the mediating roles of psychological availability and work alienation. When using AI, the mediating role of psychological availability is significantly strengthened for employees with low core task characteristics substitution compared to those with high substitution. Although AI usage generally inhibits work alienation, thereby indirectly enhancing work engagement and demonstrating a positive empowerment effect, it increases work alienation under conditions of high core task characteristics substitution, demonstrating a potential negative effect. This indicates that AI usage can exhibit a negative effect on work engagement when it involves a high degree of core task characteristics substitution. Additionally, other scholars have also pointed out the adverse effects of AI usage. For instance, Hou and Fan (2024) found that when the stress induced by the use of AI increases, employees’ work engagement decreases. Koo et al. (2020) noted that in a human–machine collaboration environment, employees who feel unable to resist the threats posed by workplace changes experience a sense of powerlessness, which in turn diminishes their work engagement. Similarly, Zhao et al. (2022) argue that when AI usage involves high levels of monitoring and work control, it weakens the positive impact of supervisor support and coworker support on work engagement. These perspectives echo our research, indicating that under certain circumstances, the use of AI can indeed have a negative impact on work engagement, partially confirming our initial hypothesis about the double-edged sword effect of AI usage on work engagement.

### 5.1. Theoretical Implications

This study has important implications for theory and research. First, this study reveals the empowerment mechanisms of AI usage, broadening the horizons in the field of organizational behavior. Existing research often views AI in the workplace as a factor that may deprive or weaken employees’ resources. For example, AI replacing certain positions can trigger employees’ concerns about job security (He et al., 2023); employees may feel dehumanized when faced with AI-driven decisions, becoming emotionless individuals (Bankins et al., 2022), which can affect their job performance and their well-being (Christoff, 2014; Lucas, 2015). However, this study finds that at the current stage, the empowering effects of AI are more significant. Based on the COR theory, this paper proposes two empowerment pathways through AI usage: AI can enhance work engagement by increasing psychological availability, and it can also boost work engagement by reducing work alienation. This finding emphasizes the role of AI in empowering employees, providing a new perspective for understanding AI usage in the workplace, and opening up new directions for future research.

Second, this study further enriches the research on the antecedents of work engagement. Existing studies mostly explore the factors influencing work engagement from either an individual perspective, such as personality characteristics (Tisu et al., 2020) and affective orientation (Burić & Macuka, 2018), or an organizational perspective, such as managerial behaviors (C. Yang et al., 2024), organizational politics (Landells & Albrecht, 2019), and organizational support (Lee et al., 2024). However, these studies have largely overlooked the new influencing factor of AI usage in the context of artificial intelligence. Based on the COR theory, this study further clarifies the impact of AI usage on work engagement, enriching the academic understanding of the antecedents of work engagement and providing a new research perspective on work engagement in the era of AI.

Third, this study contributes to the theoretical development of human–AI collaboration by introducing the moderating effect of core task characteristics substitution. It clarifies the boundary conditions for the impact of AI usage on employees’ psychological states and work attitudes. With the widespread application of AI in the workplace, human–AI collaboration has become a critical issue in organizational management. Existing research primarily explores the relationship between humans and AI from the perspectives of task allocation (Müller et al., 2017; L. Wang et al., 2020), operational safety (Matheson et al., 2019), and proximity (Sowa et al., 2021), while limited attention has been paid to the mechanisms through which human–AI collaboration affects employees’ psychological states and behaviors. By introducing psychological availability and work alienation as mediating variables, this study reveals how AI usage influences employees’ work engagement through its impact on their psychological states, providing a new theoretical perspective on the psychological mechanisms in human–AI collaboration. Furthermore, while some studies have also begun to investigate the role of job characteristics in human–AI collaboration (Berretta et al., 2023; Coupé, 2019; Verma & Singh, 2022). However, no prior research has focused on the impact of the degree of core task characteristics substitution on employees’ psychological state and work attitudes. This study introduces core task characteristics substitution as a novel variable, identifying the boundary conditions for the empowering effects of AI usage: when the core task is less substituted, AI usage has an empowering effect, but as the degree of core task characteristics substitution increases, the empowering effect of AI usage gradually diminishes. This finding not only enriches the theoretical framework of human–AI collaboration but also provides a theoretical basis for future research on optimizing human–AI collaboration models.

### 5.2. Practical Implications

This study reveals the dual impact of AI usage on employee work engagement and points out the moderating role of core task characteristics substitution in this context. Based on the research findings, organizations should adopt the following targeted measures when managing the relationship between AI and human resources in order to optimize human–AI collaboration models, enhance employee work engagement, and minimize the negative impacts brought by AI.

First, balance human–AI collaboration to enhance psychological availability. Research has found that AI applications can enhance employee work engagement by increasing psychological availability, but excessive core task characteristics substitution can weaken this positive effect. Therefore, organizations should help employees transition from passive adaptation to active empowerment. Managers can adopt the following measures. They should pay attention to employees’ mental health and help them alleviate anxiety caused by AI, for example, by establishing an employee counseling mechanism. In recognition of the necessity of “reskilling”, organizations should provide employees with training related to collaborating with AI, helping them acquire necessary new skills and enhance their personal abilities and work confidence. Organizations can also establish reward mechanisms to recognize and incentivize employees who excel in collaboration. Additionally, managers need to regularly collect employee feedback, assess the impact of AI usage on work experience, and adjust human–AI collaboration strategies accordingly.

Second, to reduce work alienation and enhance employees’ sense of belonging. Research findings indicate that AI usage generally inhibits work alienation, but in situations where the degree of core task characteristics substitution is high, AI may instead increase work alienation. Therefore, organizations need to adopt strategies to mitigate work alienation and facilitate employees’ transition from mere tool utilization to meaning reconstruction, thereby bolstering their sense of belonging and purpose. Organizations should foster a people-centric organizational culture, highlighting the core value of employees. This involves clearly communicating to employees that AI serves as an auxiliary tool rather than a replacement to prevent them from feeling displaced by technological advancements. Moreover, a clear delineation of responsibilities between humans and machines is crucial to minimizing conflicts stemming from ambiguous duties. Given that AI may weaken interpersonal work connections, organizations should encourage cross-departmental collaboration and team-building activities, fostering a high-quality interpersonal interaction environment that strengthens emotional bonds among employees. Furthermore, by setting clear work goals and emphasizing the value of employees’ contributions, organizations can help employees understand how their work impacts the enterprise and even society, thereby enhancing their sense of work meaning and mitigating the sense of powerlessness that may arise from AI substitution.

Third, it is crucial to moderately control the substitution of core task characteristics to prevent the misuse of AI. This study finds that excessively high substitution of core task characteristics can diminish the positive effect of AI on psychological usability while increasing work alienation. Therefore, when introducing AI, managers need to reasonably control its substitution level for core tasks to ensure that AI does not lead to skill deterioration or diminished professional value among employees. Specific practices include the following points. Organizations should classify AI application scenarios based on the degree of core task characteristics substitution. For tasks that highly depend on creativity, emotional intelligence, or complex decision-making, organizations should use AI as an auxiliary tool rather than the primary executor. For highly automated tasks, organizations should plan in advance for employee placement and implement accompanying programs for employee transfer or skill upgrading. Increasing job diversity is an effective way to enhance employees’ interest and engagement in work. By creating a diverse mix of tasks, employees have the opportunity to try out different responsibilities and projects, thereby increasing the richness and challenge of their work. Encouraging employee rotation across different projects and positions can broaden their horizons, enhance their adaptability, and reduce AI-induced career anxiety. Organizations also need to regularly analyze job situations, assessing the substitution ratio of AI for core tasks through job analysis and the use of AI software usage records, and flexibly adjusting job tasks based on the results to ensure that AI empowers employees rather than completely replacing their work roles, thus avoiding skill erosion and diminished core value.

### 5.3. Limitations and Prospects

This study has several limitations that set the stage for promising future studies. First, the data for this study primarily comes from employees in Chinese enterprises. Due to cultural differences, varying levels of economic development, and the diversity of management practices globally, the specificity of the current sample may limit the generalizability of the research conclusions. Especially in the context of the widespread global application of AI technology, cross-national comparative analysis is particularly crucial. Furthermore, in high-level AI usage areas such as medical diagnosis and decision-making, the implications of task substitution may differ compared to those in lower-level AI usage areas like quality control systems in production or generative AI tools in consulting work. Future research could attempt to explore from these perspectives, which would contribute to a deeper interpretation and understanding of study results.

Second, although this study adopted a multi-time-point design, the inherent limitations of self-report methods still pose a common method variance issue. To enhance the comprehensiveness and validity of this study, incorporating experimental research methods would be an effective supplement. Implementing experimental designs not only reduces the biases that self-reports might introduce, such as subjective judgment or recall errors, but also reveals more direct causal relationships between variables. Therefore, future research could consider combining experimental methods with existing research approaches to deepen and increase the credibility of the study.

Third, this study introduces core task characteristics substitution as a moderating variable to explore its influence on the relationship between AI usage and work engagement. The use of AI changes job tasks not only in terms of task content and core job characteristics but also in work processes, skill requirements, decision-making processes, work environments, and job roles. How employees perceive these changes significantly influences their attitudes and behaviors at work. Therefore, future research should examine how employees perceive various task changes and their impacts within the context of AI usage. This will enable a deeper understanding and more comprehensive grasp of the effects of AI usage.

## Figures and Tables

**Table 1:** Confirmatory factor analysis.

Model	*χ* ^2^	*df*	∆*χ*^2^ (∆*df*)	CFI	TLI	RMSEA	SRMR
One-factor model (AIA+CTCS+ PA+WA+WE)	2935.320	434	2068.584 (10) ***	0.437	0.397	0.144	0.144
Two-factor model (AIA+CTCS; PA+WA+WE)	2406.489	433	1539.753 (9) ***	0.556	0.523	0.128	0.148
Three-factor model1 (AIA+CTCS; PA+WA; WE)	1811.397	431	944.661 (7) ***	0.689	0.665	0.107	0.132
Three-factor model2 (AIA; CTCS; PA+ WA+WE)	1669.905	431	803.169 (7) ***	0.721	0.699	0.102	0.106
Four-factor model1 (AIA; CTCS; PA; WA+ WE)	1495.230	428	628.494 (4) ***	0.760	0.739	0.095	0.100
Four-factor mode2 (AIA; CTCS; WA; PA+ WE)	1074.269	428	207.533 (4) ***	0.855	0.842	0.074	0.079
Four-factor mode3 (AIA; CTCS; PA+WA; WE)	1069.930	428	203.194 (4) ***	0.856	0.843	0.073	0.080
Five-factor model (AIA; CTCS; PA; WA; WE)	866.736	424	-	0.900	0.891	0.061	0.063
CLF model	828.768	393	37.968 (31)	0.909	0.902	0.054	0.055

Note: AIU = AI usage, CTCS = core task characteristics substitution, WA = work alienation, PA = psychological availability, WE = work engagement, CLF = five-factor model + common method factor. *** *p* < 0.001.

**Table 2:** Descriptive statistics and correlations among variables.

	1	2	3	4	5	6	7	8	9
1 Gender	−								
2 Age	−0.041	−							
3 Education	0.042	−0.134 *	−						
4 Tenure	−0.089	0.741 **	−0.276 **	−					
5 AI Usage	0.001	0.215 **	0.015	0.151 *	−				
6 Core Task Characteristics Substitution	−0.05	−0.120 *	−0.069	−0.194 **	0.042	−			
7 Psychological Availability	0.09	−0.018	0.123 *	−0.008	0.254 **	−0.022	−		
8 Work Alienation	−0.081	−0.047	−0.104	−0.177 **	−0.188 **	0.339 **	−0.422 **	−	
9 Work Engagement	−0.025	−0.01	−0.055	0.001	0.235 **	−0.055	0.357 **	−0.387 **	−
M	1.452	31.541	2.097	2.731	3.367	2.341	4.113	2.511	3.366
SD	0.499	8.296	0.647	1.225	1.083	0.970	0.485	0.959	0.623

Note: * *p* < 0.05, ** *p* < 0.01.

**Table 3:** Hierarchical regression analysis.

Variables	Psychological Availability			Work Alienation			Work Engagement		
	M1	M2	M3	M4	M5	M6	M7	M8	M9
CV									
Gender	0.086	0.085	0.087	−0.098	−0.077	−0.081	−0.024	−0.026	−0.075
Age	−0.111	−0.110	−0.092	0.260 **	0.241 **	0.207 **	−0.014	−0.070	0.036
Education	0.122 *	0.119	0.084	−0.172 **	−0.129 *	−0.063	−0.058	−0.069	−0.149 **
Tenure	0.076	0.069	0.030	−0.398 ***	−0.308 ***	−0.234 **	−0.006	−0.006	−0.148
IV									
AI Usage	0.265 ***	0.267 ***	0.240 ***	−0.182 **	−0.205 ***	−0.153 **		0.252 ***	0.138 *
Mediator									
Psychological Availability									0.213 ***
Work Alienation									−0.317 ***
Moderator									
Core Task Characteristics Substitution		−0.021	−0.014		0.304 ***	0.292 ***			
Interaction									
AI Usage×Core Task Characteristics Substitution			−0.157 **			0.300 ***			
**R^2^**	0.092	0.092	0.114	0.118	0.204	0.286	0.004	0064	0.241
**∆R^2^**	0.092	0.000	0.022	0.118	0.086	0.081	0.004	0.061	0.176
**F**	5.506 ***	4.595 ***	4.993 ***	7.305 ***	11.648 ***	15.477 ***	0.271	3.761 ***	12.279 ***

Note: N = 279; * *p* < 0.05, ** *p* < 0.01, *** *p* < 0.001; all reported coefficients are standardized.

**Table 4:** Mediation effect results based on bootstrap.

Path	Effect Value	SE	95% CI
AI Usage→Psychological Availability→Work Engagement	0.032	0.012	[0.012, 0.059]
AI Usage→Work Alienation→Work Engagement	0.033	0.014	[0.011, 0.065]
Total indirect effect	0.066	0.018	[0.035, 0.105]

**Table 5:** Indirect effects at different levels of moderator.

Mediator	Moderator	Indirect Effects	S.E	Bias Corrected 95% CI
	Low Core Task Characteristics Substitution	0.047	0.016	[0.018, 0.079]
Psychological Availability	High Core Task Characteristics Substitution	0.012	0.013	[−0.006, 0.043]
	Difference	−0.035	0.015	[−0.065, −0.004]
	Low Core Task Characteristics Substitution	0.078	0.021	[0.040, 0.124]
Work Alienation	High Core Task Characteristics Substitution	−0.022	0.017	[−0.054, 0.013]
	Difference	−0.101	0.028	[−0.157, −0.048]

## Data Availability

The data presented in this study are available upon request from the corresponding author.
